# The most common skin symptoms in young adults and adults related to SARS-CoV-2 virus infection

**DOI:** 10.1007/s00403-024-02991-5

**Published:** 2024-05-31

**Authors:** Monika Zaborska, Maksymilan Chruszcz, Jakub Sadowski, Tomasz Klaudel, Michał Pelczarski, Anna Sztangreciak-Lehun, Rafał Jakub Bułdak

**Affiliations:** 1 Student Scientific Society of Clinical Biochemistry and Regenerative Medicine, Department of Clinical Biochemistry and Laboratory Diagnostics, Institute of Medical Sciences, University of Opole, Oleska 48, 45-052 Opole, Poland; 2 Department of Clinical Biochemistry and Laboratory Diagnostics, Institute of Medical Sciences, University of Opole, Oleska 48, 45-052 Opole, Poland

**Keywords:** SARS-CoV-2, COVID-19, Pathophysiology of COVID-19 in the case of skin lesions, Skin lesions in the course of COVID-19, Skin manifestations of COVID-19, Symptoms of infection SARS-CoV-2, COVID-19 dermatology, COVID-19 rashes

## Abstract

Scientists from various areas of the world indicate in their studies that skin lesions occur in the course of infection with the SARS-CoV-2 virus. This article is a review of the most frequently described cutaneous manifestations of SARS-CoV-2 virus infection and the potential pathophysiology of their development, as well as information on abnormalities in histopathological tests. The article describes the impact of some factors related to the COVID-19 pandemic on the exacerbation of chronic dermatological diseases. This work was constructed on the basis of 142 research studies, reviews, and meta-analyses, focusing on the methods and materials used in individual works as well as the results and conclusions resulting from them. Some skin lesions may be a potential prognostic marker of the course of the disease and may also be a prodromal symptom or the only symptom of SARS-CoV-2 virus infection. Stress related to the COVID-19 pandemic may exacerbate some chronic dermatological diseases. A correlation was observed between the type of skin lesions and the patient’s age. The occurrence of skin diseases may also be influenced by drugs used to treat infections caused by SARS-CoV-2. A relationship was observed between the patient’s ethnic origin and skin lesions occurring in the course of COVID-19. There is a need to further diagnose the cutaneous manifestations of SARS-CoV-2 infection and to learn the detailed pathomechanism of their occurrence in order to better understand the essence of the disease and find an appropriate treatment method.

## Introduction

The COVID-19 pandemic is one of the most important issues in recent years. It began in December 2019 in Wuhan (China) with an epidemic of pneumonia of unknown etiology [1]. At the beginning of January 2020, the pathogen was called a new coronavirus (new Corona Virus or 2019-new Corona Virus), then it was named severe acute respiratory syndrome coronavirus 2 (SARS-CoV-2) [2, 3]. The start date of the SARS-CoV-2 pandemic is clear; the problem is to clearly define its end. Taking into account the transition to SARS-CoV-2 endemicity as the end date of the pandemic and based on the vaccination/infection threshold (of 70%) as an indicator of the endemicity of this disease, this threshold has probably already been reached around the world in 2021 [4]. According to reports from the governments of selected countries, e.g., in the United States, the end of the declared state of public health emergency related to COVID-19 on May 11, 2023 is considered to be May 11, 2023 [5]. The next date for the end of the COVID-19 pandemic, announced by the Director-General of WHO, is May 5, 2023. On that day, he established COVID-19 as a disease that does not pose a threat to public health at the international level [6]. By November 9, 2023, 697,592,099 people were ill worldwide, of whom 6,936,298 died, while in Poland, by the above-mentioned date, 6,541,192 were ill and 119,708 died [7]. The virus was ultimately named severe acute respiratory syndrome coronavirus 2. SARS-CoV-2 (severe acute respiratory syndrome coronavirus-2) is the seventh human coronavirus recorded in history [8, 9]. The use of bioinformatics tools allowed it to be classified in the Coronaviridae β family (beta-coronaviruses), which also includes the SARS coronaviruses (detected in 2002) and MERS (detected in 2012) [8–10]. Despite numerous similarities in the group of betacoronaviruses, they differ in phenotype and genome. SARS-CoV-2 is 50% similar to MERS-CoV-2 and 79% to SARS-CoV, but the genome organization in the case of SARS-CoV-2 is the same as in other viruses from this group [10, 11]. Betacoronaviruses differ in the structure of the 3' end of the spike protein. In the case of SARS-CoV-2, there is 1273aa; SARS-CoV has a structure of 21493aa at the 3' end of the mentioned protein, while MERS-CoV has 1270aa [11]. SARS-CoV-2 attacks host cells using ACE-2 receptors and causes many clinical manifestations of infection, and symptoms of the disease may persist even many months after infection. According to reports, long COVID (long-term COVID-19 disease) may affect up to 65 million people [12]. Scientists from various areas of the world indicate in their studies that skin lesions occur in the course of infection with the SARS-CoV-2 virus. Depending on the studies, skin lesions occurred in 0.2% of patients in China, 2.8% in Japan, 2.45% in Thailand, 7.25% of patients in India, 20.4% in Italy, and even up to 45.65% in Spain [13–17]. There is a relationship between the occurrence of skin rashes, the geographical area where the patient lives, and his or her race. Fewer skin symptoms have been reported in Asian countries [18]. This is probably related to the ethnic diversity of human leukocyte antigen (HLA) and genetic polymorphisms in ACE-2 [14, 19, 20]. The occurrence of skin symptoms does not depend on the patient’s age; cases are observed in every age group. Importantly, cutaneous manifestations of SARS-CoV-2 virus infection may be useful in the early diagnosis of the infection and in the potential assessment of prognosis [21]. This article is a review of the most frequently described skin lesions in the course of the SARS-CoV-2 virus infection and the potential pathophysiology of their occurrence, as well as information on changes in histopathological tests (Table 1).

**Table 1:** Distribution of the literature used

Database	Pubmed, Elsevier, Google Scholar, Other		
Included	*N* = 145; *N* = 14 Record excluded during preparation of article		
Total included	*N* = 142		
Types of sources	Books *N* = 0	Articles *N* = 139	Websites *N* = 3
Year of publication	–	2014–2018 *N* = 2; 2019–2023 *N* = 137;	2023; 2023; 2023

### Pathophysiology of infection

In most cases, COVID-19 disease may be asymptomatic or may resemble a mild flu-like illness. The virus spreads through droplets, i.e., in the form of an aerosol generated during unconditioned reflexes, such as coughing and sneezing, but also during conversation [22]. Through the mentioned transmission mechanism, SARS-CoV-2 enters the target organism through the respiratory tract. It was also investigated whether the virus could attack the host through the eyeball and digestive system, but these studies concluded that this was unlikely [23]. The structure of coronaviruses is based on four glycoproteins: spike, envelope, nucleocapsid and membrane, S, E, N, and M, respectively [22, 23]. The most important structural protein of the above mentioned is the S protein, thanks to which the virus can enter the host cell. It consists of two subunits, i.e., subunit 1 (S1) and subunit (S2), connected by the S1-S2 fragment [24]. The S1 subunit is responsible for recognizing and binding to the receptor; the second subunit allows the virus to fuse its cell membrane with the cell it attacks [22]. Once it enters the organism it infects, it penetrates the host cells, thanks to the receptors located on their surface, by endocytosis [22]. The receptor recognized by SARS-CoV-2 is angiotensin-converting enzyme 2 (ACE2). The enzyme is the main element of the rennin-angiotensin-aldosterone system (RAAS); it influences water and electrolyte balance and maintains blood pressure homeostasis by catalyzing the transformation of angiotensin 2 into angiotensin 1 [25–27]. ACE 2 is anchored in the cell membrane by a transmembrane anchor, and outside the cell it has an extracellular domain to which the virus S protein directly attaches via the receptor-binding domain-RBD [25, 26]. After binding to the receptor, transmembrane serine protease 2 (TMPRSS2) cleaves the junction of the S1 and S2 subunits at the S1-S2 site. Thanks to the cleavage of the S1-S2 site by TMPRSS2, the virus can enter the attacked cell [24, 28]. Enzymes: ACE2 and TMPRSS2 are widespread in many tissues of the body, including the lungs, kidneys, colon, heart, testicles, and skin [26, 29]. The virus also attacks deep capillaries, found in the skin and subcutaneous tissue, and also targets epithelial cells, eccrine glands, and keratinocytes [1, 30]. When the virus enters the host cells, dendritic cells, macrophages, and monocytes are activated as part of the innate immune response. The phagocytic cells of the immune system adhere to infected cells and produce Interferon I (both alpha and beta), which is a specific danger signal, as well as pro-inflammatory cytokines. This action translates into the destruction of infected cells and changes in the skin [1, 31, 32].

### The most common skin changes

**Fig. 1:**
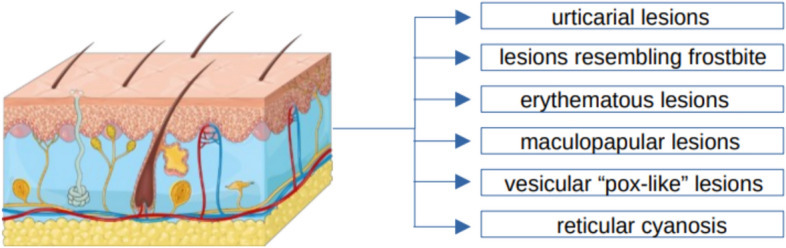
The most common skin symptoms in adults related to SARS-CoV-2 virus infection. [34–38, 45, 54, 57, 61, 74]

This part of the article describes the most common cutaneous manifestations of SARS-CoV-2 virus infection (Fig. 1). These include urticarial lesions, acral lesions resembling chilblains, maculopapular lesions, vesicular lesions resembling smallpox, erythematous lesions resembling measles, and livedo reticularis (LR) and livedo racemosa (LRC).

### Urticaria lesions

**Fig. 2:**
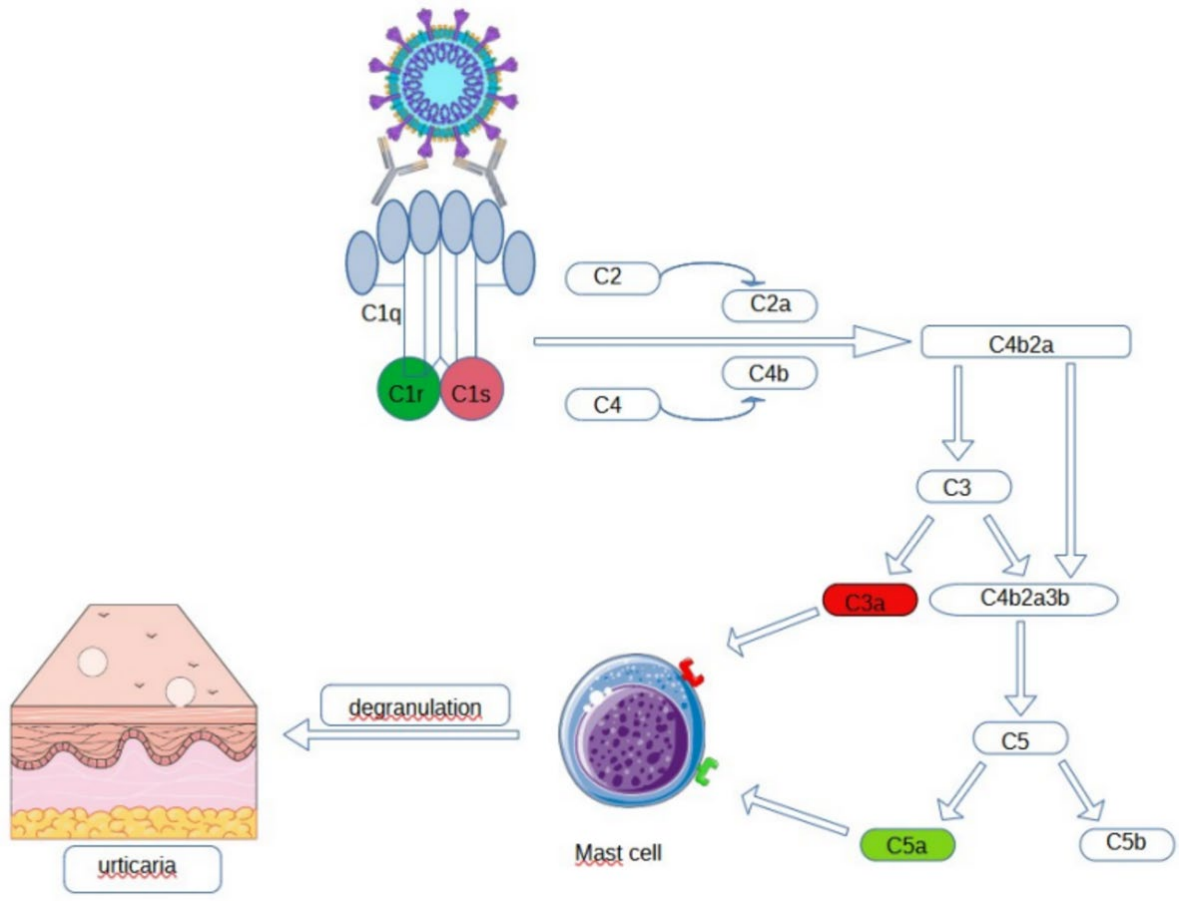
SARS-CoV-2 virus infection, the activation of the complement system, and the response of mast cells to its action. The classical pathway is activated by specific SARS-CoV-2 antibodies to the complement component C1 complex by activating the transformation of C2 and C4 molecules into active parts, which together form the C4b2a complex, activating subsequent elements of the cascade, including C3 and C5 molecules, resulting in their active parts that bind to the appropriate receptors on mast cells, causing the release of bradykinin during their degranulation [40–42]

Urticaria is characterized by the appearance of well-defined, convex urticarial wheals, pink or porcelain-white in color, accompanied by itching or burning of the skin. Its most common causes are food, drugs, infections, hymenoptera venoms, and plant pollen [33]. Urticaria lesions in the course of the SARS-CoV-2 infection lasted up to 24 h and were associated with a survival rate of 97.5% [34]. According to the AAD/ILDS report, which collected data from 52 countries, urticaria occurred in 16% of patients with skin lesions [35]. In a study conducted in Spain in which 375 cases of patients with skin lesions caused by SARS-CoV-2 virus infection were recorded, urticaria occurred in 19% of them, of which 64% were women [36]. Cases of urticaria occurred most often on the limbs and trunk, but cases of generalized urticaria or localized urticaria on the face have also been reported [36–39]. Researchers also report cases in which urticaria was a prodromal or only symptom of SARS-CoV-2 virus infection [40, 41]. The hypothetical pathogenesis of its occurrence during this virus infection is infection-induced degranulation of mast cells—activation of the classical pathway the complement system. The virus enters cells through ACE-2. Antigen–antibody complexes are deposited, resulting in activation of the complement system, degranulation of mast cells, and ultimately the release of bradykinin (Fig. 2) [42]. Some scientists suggest that the occurrence of urticaria may have a psychogenic basis, be caused by the stress associated with COVID-19, and not be related to the infection itself [43]. It is also worth emphasizing the potential drug-related etiology of this disease. Medicines used to treat COVID-19, including: antimalarial drugs (hydroxychloroquine and chloroquine), antiretroviral drugs (lopinavir, ritanavir, and darunavir), antiparasitic drugs (nitazoxanide), glucocorticoids, Janus kinase inhibitors (baricitinib), may also cause urticaria [44].

### Skin lesions resembling frostbite—*pseudo*-frostbite or pernia-like acral lesions

Skin lesions resembling frostbite, so-called pseudo-chilblains or perni-like acral lesions that appear spontaneously, without exposure to cold, are usually located on the fingers and toes and appear in the form of purple, erythematous, or purpuric papules; less often, they manifest themselves as vesicles or pustules [34, 45]. These symptoms are more often observed in children and young adults; they usually disappear without leaving scars and indicate a milder course of the disease; the survival rate among patients who experienced pseudo-frostbite was 98.7% [21, 34, 46]. Most reports of a rash resembling frostbite come from Europe and the United States, where they were among the most common skin manifestations of the SARS-CoV-2 infection and concerned the vast majority of white people. Chilblain-like lesions occur frequently in Europe and the USA and rarely in Asia [34]. These symptoms were reported and recorded in the international PedRa registry. The latest update of the report states that there were 467 cases of frostbite-like lesions among children aged 2 months to 18 years, and most of them were white men [35]. Various studies from Spain, Italy, and France describe numerous cases of patients with pseudo-frostbite [47–50]. In Asian countries, these changes practically did not occur [13, 14]. The pathomechanism of frostbite-like formation is unknown, but vascular inflammation or thrombosis, or neoangiogenesis, is suspected [49, 51]. The most widespread theory seems to be that interferon 1, during the active phase of infection, promotes the production of cryofibrinogen, which causes perniosis in acral places [45, 52]. In his research, Amit K. Maiti describes that IFIH1, which is responsible for the host’s immune response by inducing the production of interferon, responsible for cellular humoral immunity and activation of the mitochondrial antiviral system, shows racial polymorphism. This may explain why symptoms of pseudo-frostbite are most frequently observed among Caucasians [53]. Histological studies described superficial and deep lymphocytic infiltration in the dermis with a perivascular pattern and percrine enhancement, with keratinocyte necrosis [54, 55].

### Maculopapular lesions

An rash containing macules and papules was a frequently reported cutaneous manifestation of infection, predominant among adults [56]. It usually appeared within the first days of infection, was more common in women, was characterized by intense itching, and eventually disappeared spontaneously within 10 days of its appearance. The survival rate among patients with this rash was 98.2% [34, 57]. There are reports that maculopapular rash appeared after the disappearance of systemic symptoms [56, 58]. In a Spanish study, maculopapular lesions were described in 47% of patients, lasting approximately 9 days and associated with a more severe course of infection [36]. The rash correlates with the activity of SARS-CoV-2 infection; approximately 55.8% of cases occurred in the active phase of the disease, which may be directly related to the viremia phase, during which the virus enters through the bloodstream into the endothelium of vessels, where cytotoxic T lymphocytes flow, causing a rash [34]. Scientists suggest that the hypothetical cause of the maculopapular rash may be the cytokine storm of the hyperinflammatory phase, but the thesis that the rash is an adverse reaction of the body to drugs used to treat COVID-19 is widely accepted [44, 59]. However, there have been cases of patients with a maculopapular rash who were not treated pharmacologically [60]. Histologically, a superficial perivascular lymphocytic infiltrate with eosinophilia, vasodilation in the papillary and middle dermis, and moderate spongiosis of the epidermis have been reported [61, 62].

#### Vesicular “pox-like” lesions

Cutaneous manifestations of SARS-CoV-2 infection also include vesicular lesions, which occur mainly on the trunk in the form of monomorphic vesicles on an erythematous base and resemble chickenpox [63]. These lesions were revealed in 13.0% of patients included in Giulio Daneshgaran’s study, mostly after the appearance of systemic symptoms, and the average age of patients with varicella-like rash was 48.3 years [64]. In a Spanish study, vesicular lesions occurred in 9% of patients; they occurred not only on the trunk but, in some cases, also on the limbs and were sometimes filled with bloody content [36]. In turn, an Italian study described 22 patients with a vesicular rash, in whom it appeared on average 3 days after the onset of systemic symptoms and lasted on average 8 days [65]. However, there are cases in which the vesicular rash appeared before the onset of other symptoms of SARS-CoV-2 infection; therefore, its potential use in the diagnosis of infection has been suggested [64–67]. Unlike chickenpox, a rash caused by the SARS-CoV-2 virus causes mild itching [65, 68]. Scientists suggest that a potential pathophysiological mechanism for the occurrence of vesicular lesions is a cytokine storm, which is the result of excessive reactivity of the immune system or a direct effect of the coronavirus on the endothelium [69]. Histologically, vacuolar changes, hyperchromatic keratinocytes, and dyskeratosis are observed [45, 54].

#### “Morbilliform” erythematous lesions and skin lesions resembling erythema multiforme

An erythematous rash that resembles the rash seen in measles is described as a relatively common symptom of SARS-CoV-2 virus infection. Affecting up to 22% of patients, its occurrence was often associated with a higher rate of hospitalization and a more severe course of the disease, and the rash usually disappeared spontaneously and did not require treatment [45, 66]. Histopathological examination revealed spongiosis and perivascular lymphocytic and neutrophilic infiltrates, but no coronavirus spike protein was detected in the collected material. It is suggested that this rash may not be caused directly by infection with the SARS-CoV-2 virus but may have a drug-induced etiology, e.g., as a result of the use of colchicine [44, 45, 70]. Erythema multiforme is characterized by the occurrence of characteristic skin lesions with an erythematous base resembling a shooting target. The most common etiological factor is a viral infection (most often the HSV virus) or bacteria (most often Mycoplasma pneumoniae) [71–73]. Discoid lesions resembling erythema multiforme are rare lesions in the course of COVID-19, which occurred mainly in children and were associated with a mild course of the disease [21, 64].

#### Reticular cyanosis: livedo reticularis (LR) and livedo racemosa (LRC)

Livedo reticularis (LR) is a benign, usually transient skin symptom characterized by a patchy, red-blue, or purple reticulation of the skin with a cyanotic pattern. Mottled discoloration of the skin results from the spasm of blood vessels, hindering the supply of oxygen to the skin [56, 74]. According to the AAD/ILDS SARS-CoV-19 registry, 6.4% of the 1,875 cases described in patients from 52 countries were patients with LR [35]. In the majority of COVID-19 patients with reticular cyanosis, no thromboembolic complications were observed, and the disease disappeared spontaneously [45]. The term livedo racemosa (LRC) describes changes that do not go away on their own and result from pathological conditions. They occur most often on the trunk, limbs, and buttocks and are associated with greater ischemia [45, 63]. They are characterized by a more generalized and extensive arrangement, as well as a more irregular shape compared to LR [74]. These changes lasted an average of 9.4 days, most often occurred among older people, and were associated with higher mortality, reaching 20%, with a higher incidence of hospitalization and the need for mechanical ventilation, as well as a greater tendency to develop ARDS and disseminated intravascular coagulation (DIC) [56, 63, 64]. According to data from the AAD/ILDS SARS-CoV-19 registry, they concerned 2.3% of patients [35]. The pathophysiology of LR and LRC formation during SARS CoV-2 infection is not fully understood, but researchers indicate that a potential cause may be the presence of microthrombi, which are formed as a result of activation of the complement system by immunoglobulins, as well as increased concentrations of D-dimers and degradation products fibrin in the serum of patients with COVID-19 and prolonged prothrombin time [75–77]. Histopathological examination revealed inflammatory microthrombotic vasculopathy [70]. The increased tendency to clot formation and ischemia caused by the virus may be a COVID-19-specific complication, and the occurrence of livedo reticularis (LR) and livedo racemosa (LRC) may be an important prognostic marker as it correlates with the severity of the disease [78].

### Less occurring skin changes

#### Papulo-squamous lesions

Pityriasis rosea (PR) is a dermatosis with typical papulo-squamous-erythematous lesions that affects young adults and children [45, 79, 80]. A characteristic dermatological symptom of this disease is the appearance of a herald spot, which, after some time, spreads along the Langer line along the limbs and torso until the rash covers the entire skin [45, 81]. However, this change may not occur in the case of pityriasis rosea, observed in patients with SARS-CoV-2 infection. One study, which included 19 patients (mostly women, 52.6%), showed that typical PR occurred in 77.9% of the subjects, while atypical PR occurred in 21.1% of the subjects [82]. Another study described the case of a 27-year-old man in whom the course of PR was the same as in a patient not infected with SARS-CoV-2 [83]. PR is a self-limiting disease; the patient should be advised to rest in order to treat it effectively, as well as to support the patient with symptomatic treatment [81]. Researchers do not agree on the correlation between COVID-19 and PR. It has been indicated that SARS-CoV-2 may contribute to the occurrence of PR directly and indirectly [79]. It was determined that SARS-CoV-2 probably indirectly activates herpesviruses 6 and 7 (HHV6 and HHV7, respectively), which were previously dormant in PR patients. As a result, HHV6 and 7 cause PR skin lesions [84, 85]. Another factor that probably indirectly contributed to the occurrence of PR in connection with the COVID-19 pandemic was stress, which was multiplied as a result of the pandemic [79, 84, 85]. Histologically, lesions occurring in patients with PR and coronavirus infection are characterized by lymphocytic aggregates and Langerhans cell aggregates, as well as focal parakeratosis accompanied by spongiosis [45]. A group of 154 patients, including 62 women, was selected for one of the studies. The group indicated that PR most often occurs as a result of vaccination against COVID-19 (66.2%), compared to the occurrence of PR after infection (57.7%) or during infection (42.3%) [86]. One of the studies conducted on Korean patients proved that due to the COVID-19 pandemic, histopathological changes ranging from classic, i.e., dense and vigorous, to dormant infiltration were the result of SARS-CoV-2 infection. It was also shown that the occurrence of PR during the pandemic resulted in a much more frequent occurrence of pruritus in patients [87].

#### Stevens-Johnson syndrome (SJS) and toxic epidermal necrolysis (TEN)

Stevens-Johnson syndrome (SJS), toxic epidermal necrolysis (TEN), and SJS-TEN overlap syndrome are rare, severe immune-mediated cutaneous disorders with high mortality—SJS up to 29%, TEN up to 48%. They are defined as the involvement of < 10%, 10% to 30%, and > 30% of the body surface area by polymorphic lesions, respectively [88, 89]. Cases of these diseases have been described in the literature in patients with SARS-CoV-2, and Stanley and co-authors report in their study an as much as seven-fold increase in the number of SJS and TEN cases in their institution [89–91]. It is suggested that the appearance of SJS/TEN may be related to the use of drugs to treat SARS-CoV-2 infection, such as oseltamivir, lopinavir, ritanavir, hydroxychloroquine, and chloroquine, or be directly related to SARS-CoV-2 infection or vaccination against COVID-19 [44, 89].

#### Skin lesions associated with multisystem inflammatory syndrome in children (MIS-C)

During the COVID-19 pandemic, a new disease entity was identified: multisystem inflammatory syndrome in children (MIS-C). It is characterized by a hyperinflammatory response, manifesting as fever, multi-organ dysfunction, cardiovascular impairment, rash, conjunctivitis, and gastrointestinal changes [92–96]. The pathophysiology of the disease is not yet known ​[97]​. One study hypothesized that in the probable etiology of MIS-C, zonulin plays a significant role, the increased titer of which caused by SARS-CoV-2 infection causes an increase in intestinal permeability, which facilitates the passage of the virus into the blood and its attack on cells [95, 98]. Other studies indicate the probable autoimmune nature of MIS-C, which is supported by the activation of antigen-presenting cells and B/T lymphocytes as a result of infection, attacking the body’s own cells by the superantigen. It occurs close to the S1/S2 site of the spike protein, which activates immune system cells [95, 98]. MIS-C is sometimes misdiagnosed as Kawasaki disease (KD), but it is more severe because it more often affects the heart and more often causes gastrointestinal symptoms [45, 99]. Initially, 2/100,000 patients under 21 years of age suffered from MIS-C. In March 2022, the Centers for Disease Control and Prevention reported 7,459 cases of MIS-C in the US, of which 63 patients died [100]. Skin lesions occur in approximately 50–76% of patients with MIS-C, but the mechanism of their occurrence has not yet been discovered [101, 102]. In a study of 35 hospitalized children suffering from MIS-C, skin and mucous membrane symptoms lasting from several hours to several days were demonstrated by as many as 86% of the respondents. The most common symptoms included erythema on the palms, soles of the feet, and cheeks; periorbital edema and erythema; lip hyperemia; and strawberry tongue [103].

### Nail changes

Skin changes resulting from COVID-19 also affect the nails. These are rare changes that occur in patients of all ages, regardless of their origin [104, 105]. As a result of infection with the SARS-CoV-2 virus, the following have been reported: the following changes on the nails: a red crescent sign, Mees’ lines, Beau’s lines [106–108]. The red crescent sign is the most common post-COVID-19 change that affects the nails. A raised erythematous line over the distal end of the lunula is described [104, 108]. This symptom usually occurs 2–3 weeks after the SARS-CoV infection and disappears spontaneously within 2–3 months [106, 108, 109]. The red crescent symptom was also reported two days after the onset of symptoms of the SARS-CoV-2 infection [107]. Most likely, this symptom occurs as a result of an immune reaction resulting from COVID-19 directed at the capillaries of the nail bed [105, 106, 109]. One study conducted on 55 patients indicated that the red crescent symptom occurred in 32.5% of patients (fingernails) and in 2.3% of patients (toe nails) [104]. Mess lines are abnormal keratinization of the nail plate resulting from systemic diseases [107, 110]. The case of a 57-year-old Spanish patient who developed Mees’ lines as a result of the SARS-CoV-2 infection is described. As a result of treatment with lopinavir and ritonavir, the patient improved after 10 days, and the changes disappeared after 45 days [107]. Beau’s lines are transverse grooves resulting from the restriction of nail growth in its proximal matrix [111, 112]. Taking into account the occurrence of Beau’s lines in COVID-19 patients based on the distance of the Beau’s line from the proximal end nails, it can be concluded how long ago the infection occurred in patients with COVID-19 [112, 113]. This symptom may also occur as a result of Raynaud’s disease, surgery, treatment, or infectious diseases [107, 112, 113]. These lines may appear about a month after the onset of the SARS-CoV-2 infection, as in the case of a 68-year-old patient from Japan and a 41-year-old woman from the United States [113, 114]. These changes may also occur 2–3 months after infection, and their persistence depends on the length of the changes, usually lasting from 1 to 2 weeks [109].

### Chronic skin diseases extensive by stress

The SARS-CoV-2 virus pandemic has contributed to numerous sociological and economic changes, which are stressful factors for many patients [45]. The COVID-19 disease has negatively impacted the mental health of patients. An increase in the incidence of anxiety and depressive disorders has been reported [115]. In their research, scientists indicate the potential impact of psychological factors related to the COVID-19 pandemic on the severity of symptoms of chronic dermatological diseases [116–118].

### Exacerbation of psoriasis lesions

Psoriasis is a chronic, immunologically determined inflammatory disease that manifests itself regardless of age and affects approximately 60 million people around the world [119–121]. The most characteristic symptom of the disease are salmon-pink papulo-scale lesions, well demarcated from the skin, which affect the skin of the upper and lower limbs, the head, and the sacrum area [120, 122]. The occurrence of psoriasis and its severity depend, among others, on genetic factors, the patient’s age, and environmental factors [123]. Inflammation caused by COVID-19 significantly affects the course of the disease [124]. Patients suffering from psoriasis are at higher risk of severe infection with the SARS-CoV-2 virus. The fear of getting sick is an additional stress factor for them, negatively affecting the course of the disease [119]. The PsoProtect registry reported 1,652 patients with psoriasis who were infected with the SARS CoV-2 virus, of whom 13.8% required hospitalization, and 732 patients experienced worsening of psoriasis [125]. A study was conducted on a group of 374 patients from 25 countries regarding the impact of the use of biological drugs on the course of COVID-19. 93% of them recovered completely, and 21% of the patients were hospitalized. The death rate was 2%. This study showed that in patients suffering from psoriasis of varying severity (moderate and severe), the use of biological treatment resulted in a reduced risk of hospitalization related to the SARS-CoV-2 infection [126]. The online study examined the relationship between loss of income, restrictions on outdoor sports due to the COVID-19 pandemic, and changes in the severity of psoriasis. It was reported that as many as 43.7% of the respondents reported moderate or significant intensification of psoriasis [124].

### Exacerbation of atopic dermatitis (AD)

Atopic dermatitis (AD) is a chronic disease whose etiology is multifactorial and is influenced by, among others: environmental, genetic, and immunological factors. It affects approximately 10% of the population [127]. Most often, atopic dermatitis occurs in early childhood, also in infancy, and manifests itself as a rash with redness and dryness of the skin, as well as severe itching [128, 129]. Patients tend to scratch skin lesions, which leads to numerous bacterial superinfections and exacerbates the lesions, causing discomfort and negatively affecting the patients’ quality of life [127]. As a result of increased stress, the symptoms of the disease may worsen and its frequency may increase [130]. The COVID-19 pandemic is associated with social isolation and a sense of helplessness, but also with anxiety and increased stress, which potentially negatively affect the course of AD [131]. The pandemic was also associated with increased use of personal protective equipment and more frequent disinfection and hand washing, which translates into an exacerbation of AD [132, 133]. A study on the correlation between AD and COVID-19, which involved 214,206 people, showed that the risk of developing the disease is 29% higher among AD patients [134, 135].

### Telogen effluvium

Telogen effluvium (TE) is an example of non-scarring alopecia, which is associated with the transition of hair follicles from the growth phase—anagen, to the resting phase—telogen [136]. The characteristic element of TE is both acute and diffuse hair loss. It occurs 2 or 3 months after a trigger, such as physiological stress, endocrine disorders, the use of medications (such as vitamin A and heparin), nutritional disorders, or childbirth [136–138]. An increase in the frequency of TE in New York by over 400% was observed 3–4 months after the outbreak of the pandemic [136]. It is suspected that in 10% of patients infected with SARS-CoV-2, TE occurs several days before the clinical picture characteristic of COVID-19 occurs. This is particularly important due to the prediction of SARS-CoV-2 infection and the earlier implementation of COVID-19 prevention [17, 139]. In one study of 1,000 people whose TE occurred only after SARS-CoV-2 infection, 411 (73.3%) of participants experienced increased hair loss after infection, and 171 (30.5%) of those studied combined the hair, causing pain [140]. There was also a significant correlation between female gender and hair loss resulting from COVID-19; 77.3% of patients suffered from post-COVID TE [141]. A study was conducted on a group of 1,826 patients suffering from alopecia and COVID-19, The average age of the patients was 54.5 years, and the majority of the patients were men. The results of this study indicated that TE is a consequence of infection [141]. One study involving 128 patients showed a relationship between infection and the manifestation of TE symptoms. Within a month of infection, this occurred in 62.5% of respondents [142]. It is helpful to prevent TE exacerbations by providing social and psychological support to patients, thanks to which they can minimize stress, which is one of the elements that trigger TE.

## Conclusions

In connection with the examples cited in this work, special attention should be paid to the correlation between skin lesions and SARS-CoV-2 infection.

The occurrence of skin lesions such as frostbite-like lesions, erythema-like lesions, and skin lesions resembling erythema multiforme, maculopapular lesions, livedo racemosa (LRC), Stevens-Johnson syndrome (SJS), and toxic epidermal necrolysis (TEN) may predict the course of infection. In turn, the appearance of livedo reticularis (LR) or urticarial lesions may indicate a mild course of the disease and a probably good prognosis.

Some changes, such as hives, "pox-like" vesicular lesions, telogen effluvium, and exacerbation of atopic dermatitis, are the first symptom of infection and can be used in diagnostics as an indicator of COVID-19, facilitating an accurate diagnosis.

Generally, cutaneous manifestations of SARS-CoV-2 virus infection affected patients in all age groups, but a correlation was observed between the type of skin lesions and the patient’s age. Some skin lesions have an increased tendency to occur in children and young adults. These include perni-like acral lesions, discoid lesions resembling erythema multiforme, and pityriasis rosea. Characteristic for this age group were rashes occurring in a newly classified disease: multisystem inflammatory syndrome in children and young adults (MIS-C). In turn, skin lesions such as maculopapular lesions, "pox-like" vesicular lesions, or livedo racemosa (LRC) were more common in older people (Table 2).

**Table 2.** Division of skin manifestations of SARS- CoV- 2 infection according to age of occurrence [21, 45, 56, 64, 79, 80]

Common cutaneous manifestations of Sars-Cov-2 infection occurring in children and young adults	Common cutaneous manifestations of Sars-Cov-2 infection occurring in adults
Perni—acral lesions	Maculopapular lesions
Discoid lesions resembling erythema multiform	“pox-like” vesicular lesions
Pityriasis rosea	Livedo racemose

In the case of some diseases, drugs used to treat COVID-19 play an important role in their exacerbation or occurrence. The above group includes: urticaria, morbilli-like erythematous lesions, Stevens-Johnson syndrome (SJS), and toxic epidermal necrolysis (TEN).

The deterioration of the mental health of patients with chronic dermatological diseases, such as atopic dermatitis or psoriasis, as well as the use of personal protective equipment, may exacerbate these diseases.

Research on the ethnic diversity of human leukocyte antigen (HLA) and genetic polymorphisms in ACE-2 should be expanded, which is the probable cause of differences in the occurrence of cutaneous manifestations of COVID-19 depending on the geographical area where the patient lives and his or her race.

There is a need for further research on the cutaneous manifestations of SARS-CoV-2 infection and the detailed pathomechanism of their occurrence in order to better understand the essence of the disease and find an appropriate treatment method.

## Declarations

### Data/code availability

Data/code sharing are not applicable to this article as no datasets or code were generated and/or analyzed during the current study.
